# Stratification of stakeholders for participation in the governance of coastal social-ecological systems

**DOI:** 10.1007/s13280-023-01844-1

**Published:** 2023-03-23

**Authors:** Louis Celliers, Lena Rölfer, Nina Rivers, Sérgio Rosendo, Meredith Fernandes, Bernadette Snow, María Mãnez Costa

**Affiliations:** 1 Climate Service Center Germany (GERICS), Helmholtz-Zentrum Hereon, Fischertwiete 1, 20095 Hamburg, Germany; 2 Faculty of Sustainability, Social-Ecological Systems Institute (SESI), Leuphana University Lüneburg, Lüneburg, Germany; 3 Institute for Coastal and Marine Research (ICMR), Nelson Mandela University, A Block, Ocean Sciences Campus, Gommery Ave. Summerstrand, PO Box 77000, Gqeberha, 6031 South Africa; 4 Interdisciplinary Centre of Social Sciences (CICS.NOVA), Faculty of Social Sciences and Humanities (FCSH), Nova University of Lisbon (UNL), NOVA FCSH, Colégio Almada Negreiros, Campus de Campolide, 1070-312 Lisbon, Portugal; 5 One Ocean Hub, Law School, University of Strathclyde, Lord Hope Building, 141 St James Road, Glasgow, G4 0LT UK

**Keywords:** Environmental management, Ocean and coastal governance, Social-ecological systems, Stakeholder agency, Stakeholder analysis

## Abstract

Knowledge co-production has become part of an evolution of participatory and transdisciplinary research approaches that are increasingly important for achieving sustainability. To effectively involve the most appropriate stakeholders there is a need for engagement and increasing prominence of stakeholders in environmental management and governance processes. The paper aims at developing and testing a methodology for stratifying stakeholders by (i) classifying organisations involved in coastal and ocean governance by their agency, and (ii) grouping them into organisational archetypes for representation and selection in research processes. Agency was measured by the three dimensions of scale, resources, and power. Each dimension was further elaborated as a set of indicators. The methodology is applied in the context of a research project set in Algoa Bay, South Africa. The stratification of organisations enabled the research team to gain a better understanding of the stakeholder landscape of organisational agency, and thus identify the most relevant stakeholder with which to engage. The use of a hierarchical cluster analysis identified five organisational archetypes in relation to ocean and coastal governance in Algoa Bay. The methodology used in this study proposes an informed and intentional approach to create the conditions under which the co-production of and participation in research processes can take place.

**Supplementary Information:**

The online version contains supplementary material available at 10.1007/s13280-023-01844-1.

## Introduction

The sophistication of engagement with stakeholders as a fundamental part of environmental management and governance processes is an increasingly important topic of research (e.g., Burdon et al. 2019; McKinley et al. 2021). Stakeholders are those who have something to win or lose in the governing process (Jentoft 2007), or by the urgency of their concerns, the legitimacy of their interests, and the power they hold (Buanes et al. 2005). Stakeholders are also defined relative to a particular issue which is time- and site-specific (Glicken 2000).

Stakeholders are active participants in knowledge co-production and are often described as “owners” or initiators of the process for which research outputs are intended to create societal impact (Turnhout et al. 2020; Vollstedt et al. 2021; Strand et al. 2022). Knowledge co-production has become part of an evolution of participatory and transdisciplinary research approaches that are increasingly important for achieving sustainability (Mach et al. 2020; Norström et al. 2020). Knowledge co-production processes are interactive and engage both scientific actors and non-academic stakeholders (Scott et al. 2021; Rölfer et al. 2021; Rivers et al. 2022). Actors from outside the academic spheres are recognised for contributing legitimate and often unconventional forms of knowledge and expertise that are increasingly seen as indispensable for solving societal problems (Polk 2015). Some authors describe co-production as one of the most important ideas in the theory and practice of knowledge and governance for global sustainability (Miller and Wyborn 2020).

Legitimate stakeholders are often poorly stipulated or specified in many research projects (Lavery 2018). For example, in the context of climate change adaptation, marginalized stakeholder groups tend to be more vulnerable to climate change, but at the same time are less represented in participatory processes (Thomas et al. 2019). In the absence of a process for a careful selection based on the objectives of the effort, there is a danger of only engaging the “usual suspects”. These are often small but vocal groups of stakeholders who are already widely engaged not only in research but also in policy and practice. While often convenient for research projects, the continued engagement with willing and available stakeholders may also reinforce the marginalization and exclusion of groups whose voices are rarely heard, thus limiting social learning with potential ethical questions (Stringer et al. 2006).

The rationale for the selection of stakeholders to engage in knowledge co-production in research processes is therefore increasingly important. Who are the right stakeholders involved at the right time and in the right way?—so-called “proper and pertinent stakeholders” (Ahmadi et al. 2019). Stakeholders, as active participants, must be able to act in some meaningful way. For stakeholders to be able to act (i.e., become actors) they need to have *agency*. Generally, agency can be defined as the capacity of individuals and collective actors to change the course of events or the outcome of processes (Pattberg & Stripple 2008; Otto et al. 2020). Some of the key elements that may enable agency include: access to resources, discourses and networks of actors (Duygan et al. 2019, 2021); power (Morrison et al. 2019); and, system roles, power and influence, alignment to the problem, and transformational potential (Lyon et al. 2020). Agency is therefore an important characteristic of the ability of stakeholders to be active participants in knowledge co-production and resulting governance processes.

Research projects that employ transdisciplinary knowledge co-production should therefore be cognisant to include stakeholders that can act to contribute to governance objectives. While stakeholders are often classified by administrative level (local to national), organisational type (e.g., governmental, non-governmental), or sector, such classification pays insufficient attention to their actual agency to act in governance processes. Instead, characteristics that constitute agency are more diverse and create a mosaic of stakeholders that is dynamic relative to the issues and objectives of co-production and governance. This is complex in all contexts, and particularly so in coastal social-ecological systems (SES) because of the numerous stakeholders with diverse interests, the dynamic nature of the environment, and the often overlapping and even conflicting legislation and policy (Pasquier et al. 2020).

This paper builds on previous work by Celliers et al. (2007) and other methodologies with which to analyse and select stakeholders (Mitchell et al. 1997; Reed et al. 2009; Lyon et al. 2020). The paper aims at developing and testing a methodology for stratifying stakeholders by (i) classifying organisations involved in coastal and ocean governance by their agency, and (ii) grouping them into organisational archetypes for representation and selection in research processes. The proposed methodology is applied in the context of a co-production process (climate services for coastal adaptation) in Algoa Bay, South Africa. This methodology was tested during the COVID-19 pandemic and adapted for limited direct engagement with stakeholders while still resulting in a transparent selection of stakeholders in engagement processes relative to a research objective.

## Methodology

### Study area

Algoa Bay is locally governed by the Nelson Mandela Bay Municipality (NMBM) consisting of the city of Gqeberha as well as the major towns of Kariega and Despatch in the Eastern Cape province of South Africa. Algoa Bay is an important social and economic hub driven by several automotive supplier companies, two ports and also the only international airport in the Eastern Cape. Algoa Bay is a popular tourist destination, especially for water sports and the nearby Greater Addo Elephant National Park and its recently promulgated Marine Protected Area (MPA; May 2019).

Since the demise of Apartheid, South Africa promulgated new or updated legislation to align with its post-Apartheid constitution. This suite of legislation includes National Environmental Management Act which also includes legislation for Integrated Coastal Management (ICM), Marine Spatial Planning (MSP) and Marine Protected Areas (MPA). The ICM Act, for example, creates a nested system of coastal management from national to local government (Celliers et al. 2013). This interplay of a diversity of ecological features and resources, legislation, management approaches, and social-economic aspects make Algoa Bay a representative case study of a complex coastal SES, and for testing the methodology. It also creates a multi-layered stakeholder landscape that is diverse and dynamic, making engagement challenging.

The proposed stakeholder stratification methodology was developed as part of the Cities and Climate Change in Coastal Western Indian Ocean (CICLICO) research programme. The project adopted a knowledge co-production approach and research activities included an assessment of governance performance for climate change adaptation in Algoa Bay, a social network analysis, and co-production of climate services. The stratification of stakeholders was critically important due to the numerous and diverse stakeholder composition in Algoa Bay relative to the research objectives. At the outset of the project, there was a prevalence of “stakeholder fatigue” that influenced the overall engagement strategy of the project, and the subsequent demand for a much more focused engagement with key stakeholders in the co-production of climate services (i.e., municipal officials).

### Identifying stakeholders

**Fig. 1:**
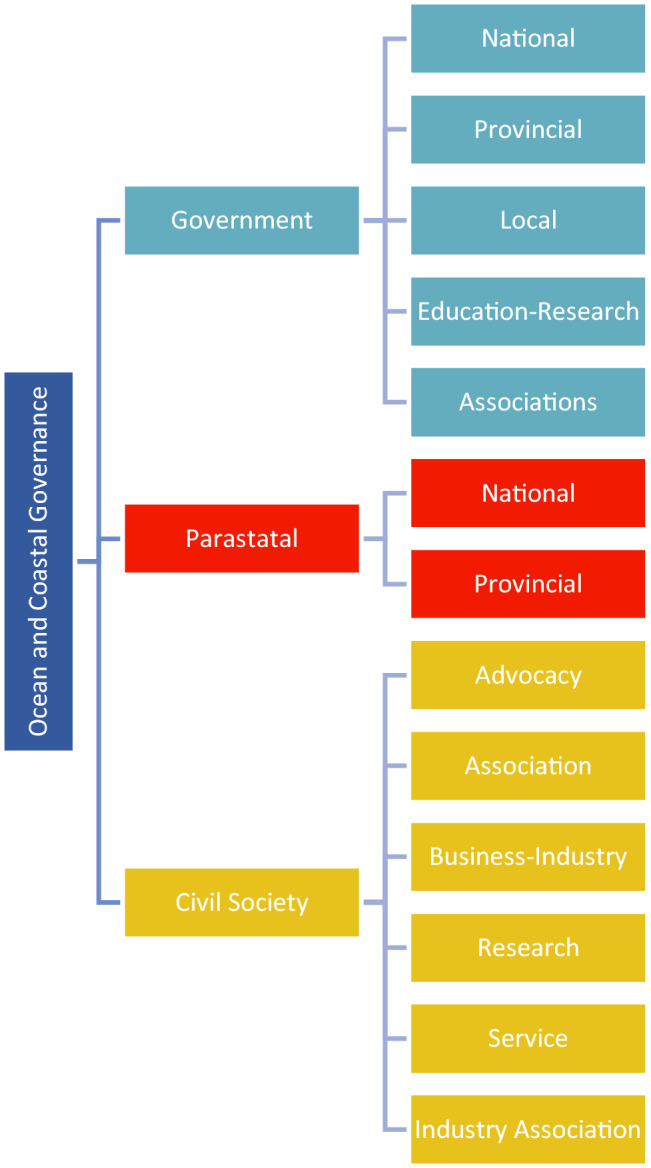
Organisational type and sub-type of organisations that have a role or interest in ocean and coastal governance in Algoa Bay, South Africa

The stakeholders in coastal and ocean governance of Algoa Bay were initially classified by organisational type to understand the complexity of representation with regards to their role and interest in ocean and coastal governance in the Bay. Three primary organisational types were identified, namely government, parastatal (semi-state) organisations, and civil society organisations (Fig. 1). A second classification provided more elaboration on the organisational sub-types.

An initial selection of stakeholders included any organisation that had an apparent interest in coastal and ocean governance. Stakeholders were identified from a review of the literature and online resources, Environmental Impact Assessments, provincial, and local coastal working groups. Organisations included were from local, provincial, and national government authorities, community organisations, environmental organisations, development groups, special interest groups, trade unions, landowners, sport and recreational bodies, tourism organisations, and business associations. This initial list of stakeholders was subsequently augmented through chain referrals from known stakeholders (Leventon et al. 2016).

### Dimensions of agency

**Fig. 2:**
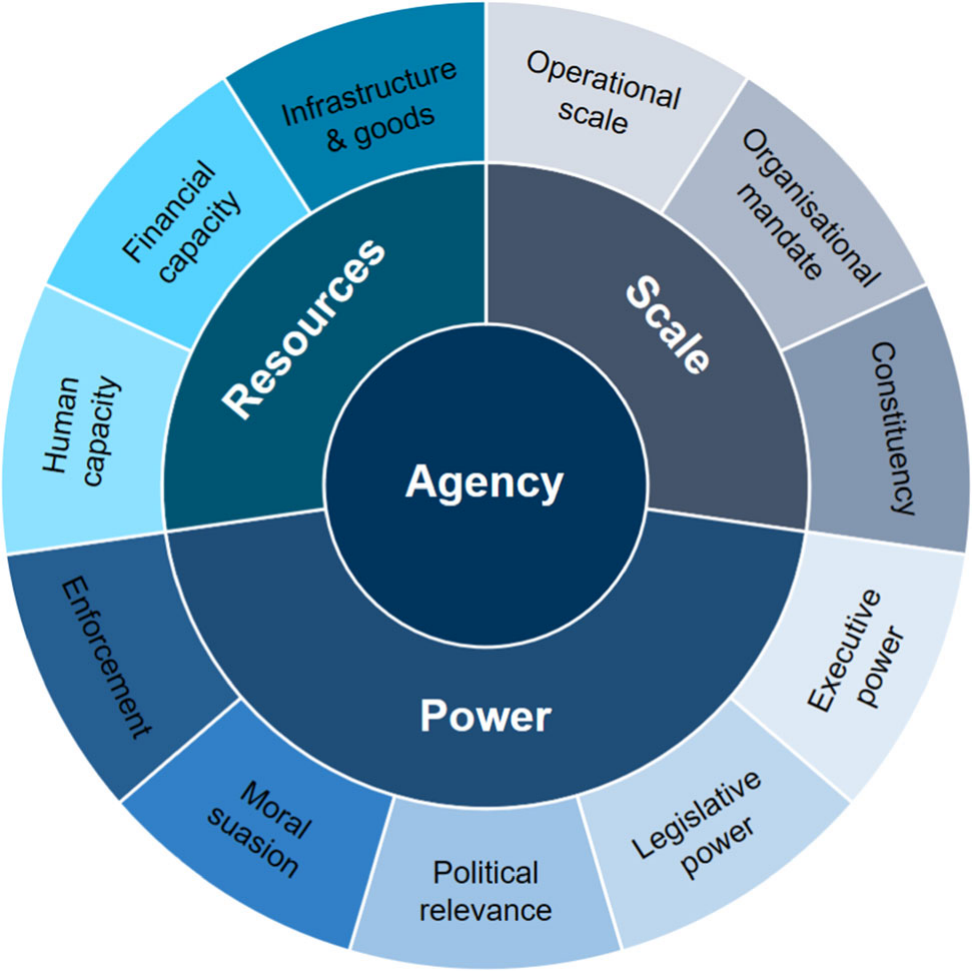
Dimensions of agency redefined for the specific context of this study

The dimensions of agency used in the stratification of stakeholders were *scale*, *power*, and *resources* as previously proposed by (Celliers et al. 2007), and redefined for the specific context of this study (Fig. 2). Each dimension of agency was further elaborated as a set of indicators for the different dimensions. This was based on the work of Celliers et al. (2007), and previous experience within coastal governance and knowledge of the contributing elements for effective governance.

#### Dimension 1: Scale

The scale or level at which a stakeholder operates is an aggregate of spatial and functional parameters, and is critically important in this context (e.g., Ernoul and Wardell-Johnson 2013; Pereira et al. 2020). Scale normally refers to geographic or spatial extent, while level refers to different administrative units often linked to spatial scale, i.e., municipalities or local government. Each stakeholder operates in a defined *operational (often spatial) scale*; some stakeholders are locally based, some are provincial (regional), and some function at a national scale. The scale of a stakeholder’s operation will have a direct bearing on their frame of reference and the perspective they bring to the network.

The function of an actor within the network is determined by a concept of their “charter” or *mandate,* which can be bestowed as legislative, political, or operational objectives. The charter restricts interaction with neighbouring stakeholders and the legitimacy with which a stakeholder engages with issues raised within the Algoa Bay system. Another interesting aspect of scale is the influence of the *representativeness* of the organisation; this means that representativeness refers to the *constituency* represented by the stakeholder. In other words, an organisation may present or demonstrate interest at a large spatial scale but by a small constituency. Conversely, for example, a professional society could have a limitation in operational scale but represent many persons directly affected by the policy issues under consideration, e.g., an organized group of many different stakeholders.

#### Dimension 2: Power

The role and influence of power on relationships between organisations is a key dimension of the agency of organisations. Power is also unavoidable and should be discussed openly (Turnhout et al. 2020). It has also been highlighted as an important feature for transforming to higher degrees of resilience and sustainability (Olsson et al. 2014; Barnes et al. 2020). For the purposes of the present analysis, we have chosen to consider power as a function of political relevance, legislative power, executive power, moral power or suasion and the power to enforce decisions or regulations.

*Political relevance* is the extent to which the institution has a specific political role to play in the policy issues being dealt with (e.g., Nightingale 2017). Some institutions are part of the public sector and have specific political roles to fulfil; some individuals might be elected officials with political scripts to follow, while some may have an ostensibly ‘politically neutral’ position, such as professional bodies or academic and research institutions.

*Legislative power*, is the ability to create, modify and repeal laws that govern society including the power to make rules and regulations, both formal and informal (e.g., Martino et al. 2019). Legislative power will also differ between organisations. Some organisations, such as organs of civil society, may have no legislative power, while organs of state (local, provincial, and national) may have considerable power in their sphere.

*Executive power* refers to the capacity and mandate to make decisions (e.g., the distribution of executive power between levels; Celliers et al. 2015). Some organizations will have been delegated power by the government (national, provincial, or local) to make decisions that affect all citizens within their area of jurisdiction e.g., in the Algoa Bay case national government delegating decisions to the conservation agency SanParks. Other organizations may only be able to make decisions that are binding on their members.

Some organizations will have *moral power* or *moral suasion* which may or may not be in proportion to their scale. This power is the power to speak with authority on a topic and to bring to the discussion opinions and examples that may persuade others to follow the stakeholder’s lead. It is expected that if the issue being discussed is of a scientific nature a research organization with a reputation for excellence will exert a large degree of influence simply because of the weight of its moral authority. Moral power is the ability to persuade, i.e., where people or groups that may hold little practical power manage to influence situations in a positive or negative direction through persuasiveness (Bos et al. 2020; Lyon et al. 2020).

Some organizations are also likely to have some degree of *enforcement power*, i.e., the power to compel either other members of the organization, or members of the public, to comply with decisions made by the stakeholder (e.g., Tosun 2012). This may be a constitutionally created power such as that enjoyed by the police force, or it may be a power assented to by virtue of membership in a group, e.g., a fishing club must enforce its constitution and conditions of membership.

#### Dimension 3: Resources

Each of the organizations that make up the stakeholder constellation will also be endowed with varying amounts of capital: financial capital, human capital, and infrastructure in the form of equipment and other physical assets. The *financial capital* of an institution is an important factor in determining the extent of the human, infrastructural and other resources available to the institution. Financial resources are often a limiting factor in determining priorities among the different policy issues with which an organization must deal. For example, resource limitations from central government are often a barrier to long-term climate adaptation (Porter et al. 2015) and a lack of resources also influences engagement with society (Baker et al. 2012).

The *human capital* that an organization has at its disposal is a function of the number of people it can deploy on a policy issue and the extent of the knowledge-base that those people possess. Sufficient in-house human or technical capacity or access to external relevant expertise makes the use of scientific information for management more likely (Lemos et al. 2012), and mainstreaming climate change adaptation is expected to be challenging because of existing strains on resources and capacity in many developing countries (Pasquini et al. 2013).

Finally, *infrastructure* (e.g., communication, mobility) is a further component of the resource dimension and includes such things as vehicles, boats/ships, telephones, offices, and equipment as well as special hardware and other physical assets. The extent of the infrastructure available to an organization, both in terms of quantity and quality, affects the extent to which an organization can quickly and easily communicate, respond to issues, engage in research, and access other members of the network or other resources.

### Evaluation of agency

Dimensions of agency were elaborated in an evaluation framework that consisted of indicators, evaluation criteria, description, and a scoring system (see Supplementary Table S1–S3). Critical design principles of the evaluation framework included the ability to: (a) apply the framework remotely due to the inability to meet in person during the COVID-19 pandemic; and (b) simplicity of indicators and scoring categories to allow for a fast and accurate assessment by experts or expert panels using publicly available sources of information such as organisational websites or annual reports.

The first step of applying the evaluation framework was an assessment of the organisation by three experts working independently from one another. The second step was a consensus process where the expert panel debated scores, fact-checked assumptions, and agreed on final scores. The three expert evaluators were knowledgeable about the social, ecological, and economic context relative to the coastal and ocean area of Algoa Bay. Scoring (and scoring validation) of organisations were originally intended for a broader stakeholder panel including the expert evaluators, but under COVID-19 lockdowns, continuous and convenient access to such a stakeholder panel was not possible.

### Data analysis

The scores for the different dimensions of agency were calculated as a normalized aggregate of the indicators for each organisation. An overall score for each organisation was defined as ‘agency’, which was calculated as a normalized aggregate across all indicators. The normalized scorings range from 0 to 1 with 1 indicating the highest score. An agency of 1 would be an organisation that has a physical presence in Algoa Bay with a high institutional mandate and constituency, which is highly resourced and has the highest power e.g., Nelson Mandela Bay Municipality.

A hierarchical cluster analysis (HCA) was performed using the statistical software R (R-Core-Team 2021) to identify clusters of organisations with similar scorings for the indicators within clusters, but distinct from other clusters. An agglomerative bottom-up approach applying the ‘complete-linkage clustering’ method was used, which forms clusters of organisations based on the maximum Euclidian distance (dissimilarity) between different clusters. The dissimilarity clustering approach was chosen to identify archetypes that are distinct from each other. Using this approach, the agglomerative coefficient was 0.86, meaning that 86% of the variance are explained by the clustering. The optimal number of expected clusters (*k*) was identified by the Sum of Squares of the dataset and set to *k* = 5 at a distance (similarity) of 5.8. A dendrogram was plotted showing all 113 institutions assigned to groups 1–5 accordingly (see Supplementary Fig. S1).

## Results

The methodology for stratifying stakeholders resulted in classifying organisations involved in coastal and ocean governance by their agency to act in governance processes and grouping them into organisational archetypes for representation and selection in research processes.

### Classification by agency and organisational type and sub-type

**Fig. 3:**
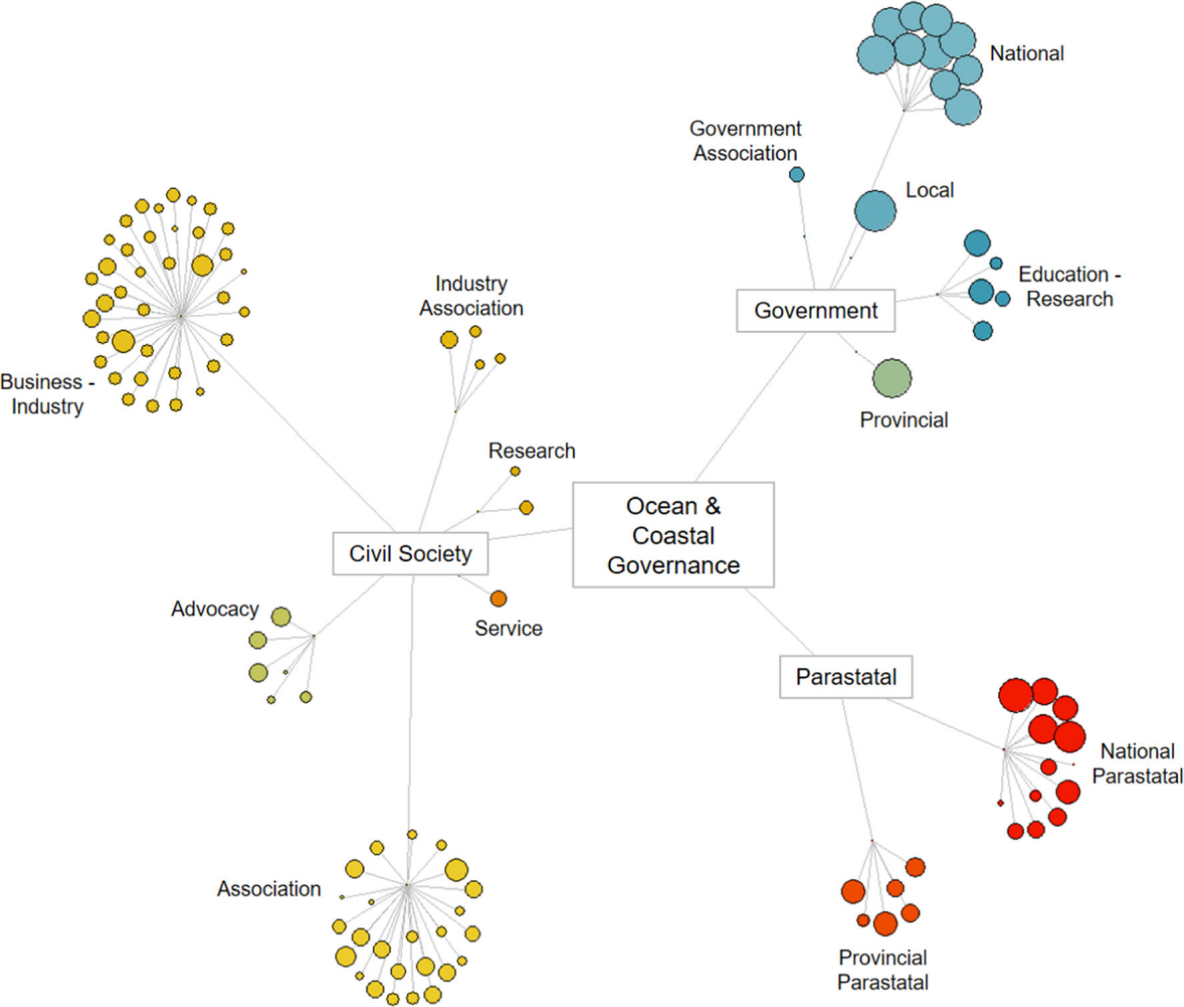
The network of 113 organisations involved in aspects of ocean and coastal governance in Algoa Bay classified by their organisation type and sub-type. Each coloured circle represents an organisation, and the circle size shows its degree of agency in the governance system (e.g., organisations with larger circles have higher agency)

From the initial desktop analysis, 113 organisations were identified: 18 from government, 19 parastatal and 76 civil society organisations (Fig. 3).

The indicators of agency show that some of the organisational groups inherently have greater agency, e.g., national government and parastatals. The organisational agency is generally lower in civil society (< 0.3) but there are more organisations (*n* = 76; Table 1). Civil society organisation indicators for power score generally low, as opposed to that of government. Government sub-groups show higher agency for local government (municipality), followed by provincial and then national. This is primarily driven by a similar trend in resources. It is worth noting that the type of resources for these sub-groups are probably very different, i.e., strategic, and high-level planning resources at the national level (i.e., data and information), as opposed to tactical and operational support for local government (i.e., bulldozers and local knowledge).

**Table 1:** Arithmetic mean scores across indicators of the dimensions of scale, resources, and power, and aggregated as agency, of organisational types and sub-types involved in aspects of ocean and coastal governance of Algoa Bay, South Africa

Organisational (Sub-)type	*n*	Scale	Power	Resources	Agency (mean) ± SD
Civil society	76	0.48	0.10	0.36	0.27 ± 0.08
Advocacy	6	0.35	0.13	0.43	0..27 ± 0.13
Association	25	0.44	0.16	0.36	0.29 ± 0.09
Business-Industry	38	0.56	0.04	0.35	0.27 ± 0.07
Industry Association	4	0.31	0.21	0.28	0.26 ± 0.06
Research	2	0.17	0.18	0.47	0.25 ± 0.07
Service	1	0.67	0.15	0.33	0.34
Government	18	0.69	0.56	0.65	0.62 ± 0.19
Association	1	0.44	0.25	0.28	0.31
Education-Research	5	0.53	0.18	0.67	0.41 ± 0.12
Local	1	0.89	0.75	1.00	0.86
National	10	0.75	0.74	0.62	0.71 ± 0.07
Provincial	1	0.83	0.75	0.89	0.81
Parastatal	19	0.49	0.30	0.52	0.41 ± 0.17
National	13	0.45	0.33	0.52	0.41 ± 0.20
Provincial	6	0.56	0.23	0.54	0.40 ± 0.09
Grand total	113	0.52	0.21	0.43	0.35 ± 0.17

*SD* Standard Deviation of scoring between organisations belonging to the same organisational sub-group

### Organisational archetypes

The use of the hierarchical cluster analysis enabled the identification of organisational archetypes in relation to their agency to act in ocean and coastal governance in Algoa Bay. The analysis (see dendrogram, Supplementary Fig. S1) resulted in the definition of five groupings of organisations that shared common characteristics of the individual dimensions and indicators of agency.

**Fig. 4:**
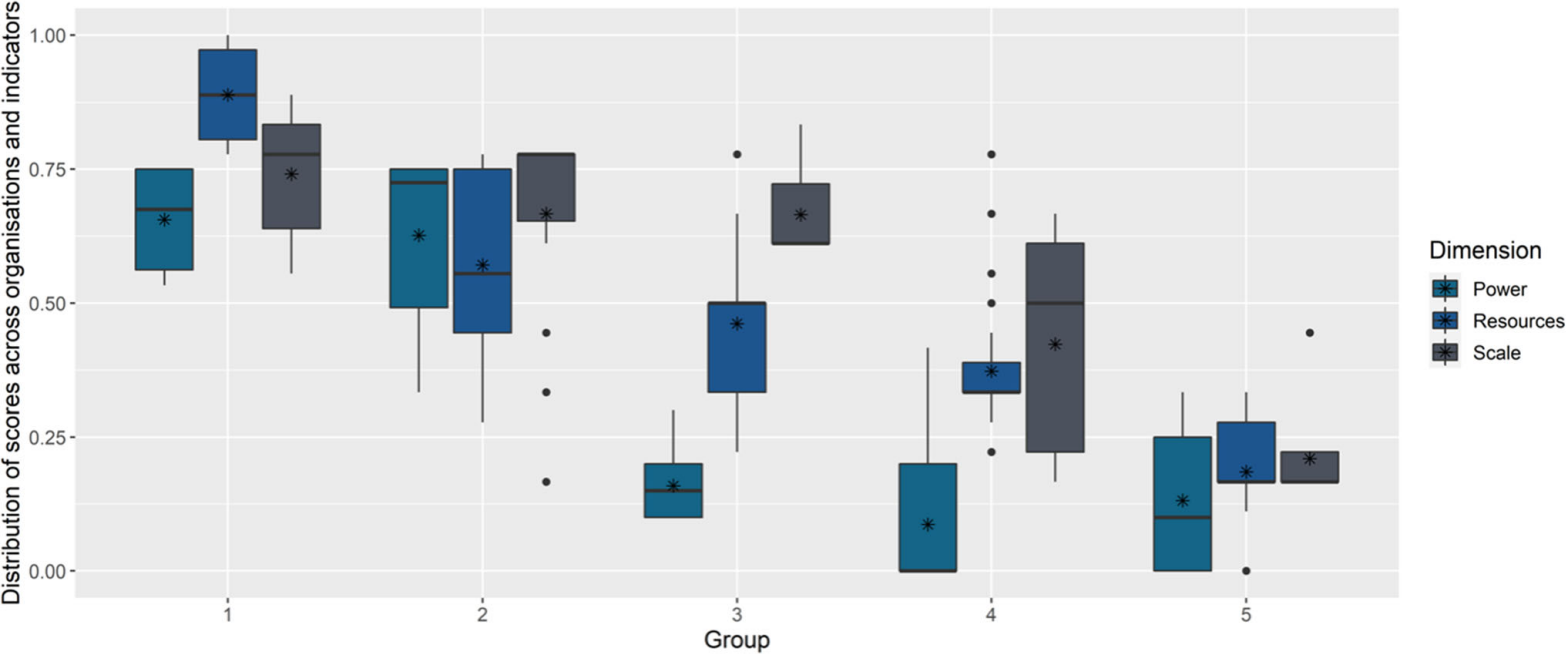
Distribution of scores for power, resources and scale across organisations and indicators for five organisational archetypes involved in aspects of ocean and coastal governance in Algoa Bay, South Africa. Boxes show 75th percentiles of distribution; stars indicate the arithmetic mean score per dimension and organisational archetype; dots visualize outliers

The hierarchical clustering of organisations by agency was further interpreted through the analysis of the statistical summaries for each of the grouping (Fig. 4). These summaries were combined with the known organisational mandate of the members of the groupings, which resulted in stakeholder archetypes stratified by similarities of three indicators of agency (Table 2). For example, organisations in group 1 are characterized by comparably high scores for power and resources and are represented at different scales (see outliers; Fig. 4). By looking at the organisational types in this group (mainly governmental) the archetype “plans and planning” was proposed.

**Table 2:** Description of organisational archetypes (resulting from HCA groupings) of organisations involved in aspects of ocean and coastal governance in Algoa Bay, South Africa

Group no	Archetype	Description
1 (*n* = 6)	“Get it done”	This is a small group of organisations with high agency. They have high measures of available resources and operational scale, and high measure of power. These organisations can act locally, and to implement decisions on local issues, in a relatively short period of time. Management actions are directly related to ocean and coastal governance, and the impact of such actions will be experienced by many stakeholders in the system. These organisations have direct authority over implementation and a significant control of policy-implementation processes. These organisations must be included in most science-society engagements related to developing the knowledge-base for local decision-making, e.g., climate change adaptation, biodiversity protection. The Nelson Mandela Bay Municipality (NMBM) is a good example of a representative organisation
2 (*n* = 14)	“Plans and planning”	This relatively large and diverse group of mainly government institutions are mostly thematically or sectorial focussed, i.e., transport, minerals and energy, environment. These organisations have substantial power but mostly brings this to bear through national policy and legislation. There are no locally based organisations in this archetype group but their role is clear with regards to medium- to long-term strategic planning in the ocean and coastal governance domain. This group is well-resourced in terms of human capacity and access to data and information. While they are scoring high for agency overall, it can be argued that they have substantially less agency compared to Group 4. Selection of participation from this group is largely dependent on their sectoral interest and the objective of the governance/stakeholder processes under consideration. The national Department of Environment, Forestry and Fisheries (DEFF) is a good representative organisation of this archetype
3 (*n* = 29)	“Little by little”	This is a large group of organisations who are low in power, but present and active in Algoa Bay. They are relatively well-resourced and operate at the Bay-scale. There are overlaps with other groups (Group 2 in particular), but this group is very relevant to focussed activities in the ocean and coastal space of Algoa Bay. With their relative high level of resources and their local presence and agency, they are important and relevant actors for local decision-making. The South African Environmental Observation Network (SAEON) and the Nelson Mandela University (NMU) are good representative organisations of this archetype
4 (*n* = 9)	“On the margin”	This small group of organisations contribute mostly data and information without authority and without being physically based or operating specifically in Algoa Bay. Low power and physically distant, this archetype can make focussed input to participation processes but may also be omitted due to the challenge of engaging from a distance. Internally, members of this archetype are also very diverse. The Oceanographic Research Institute (ORI), or the Water Research Commission (WRC) are good representative members of this archetype
5 (*n* = 55)	“Vocal and insistent”	This is a large, internally diverse group of organisations that typically score low on all measures of agency. Their physical presence in Algoa Bay makes them relevant stakeholders and their collective interest and agency makes their contribution in participatory processes important and bordering on critical. Even though their operational scales may be small, i.e., conservancy of an area within the larger Algoa Bay area, they are important for latent/dormant power, and the vulnerability of their members. A number of these organisations, given enough motivation and concern, can bring to bear power in the form of moral suasion e.g., fishing companies, community-based organisations. This is also the most difficult archetype to involve in participation processes due to their diversity of interests, motivation, capacity, vulnerability etc. This archetype can easily be to split in smaller sub-groupings. Identifying a typical organisation from this group is difficult due to the high degree of diversity of members but an example could be local NGOs, civil society advocacy groups etc

**Fig. 5:**
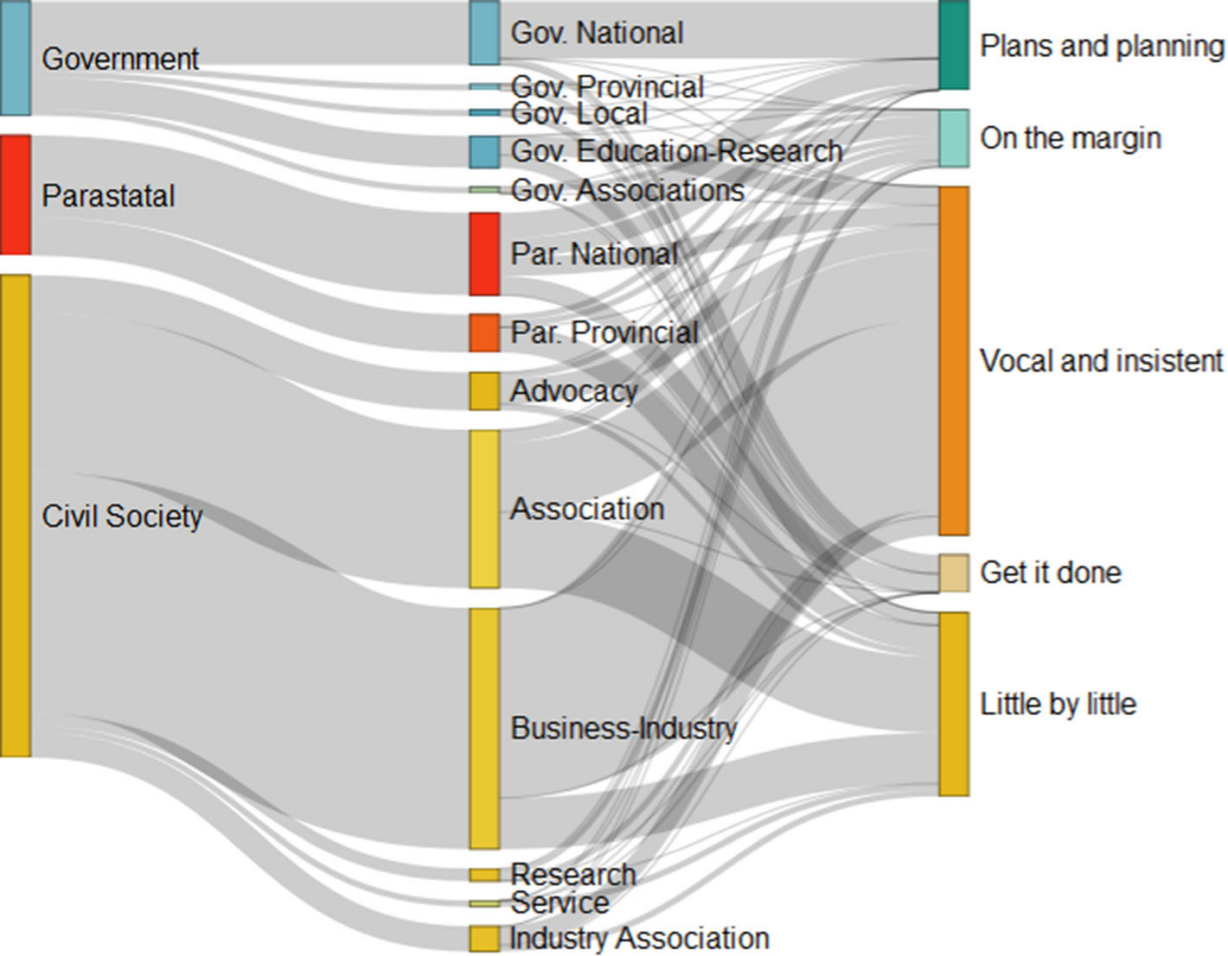
Sankey diagram visualizing the proportion of organisational types and sub-types to organisational archetypes

For providing a better overview of the stakeholder landscape in the case-study area of Algoa Bay, the contribution of the different organisational types and sub-types to the resulting organisational archetypes based on their scoring for different indicators of agency is visualized in Fig. 5.

## Discussion

With the increasing importance of knowledge co-production between researchers and non-academic actors, an informed approach to stakeholder and public engagement in research processes can improve the outcomes of co-production. Especially in complex coastal SES, where effective governance is an important driver for achieving sustainability objectives, the participation of the ‘right’ stakeholders is essential. The approach used in this study proposes such an informed and intentional approach to create the conditions under which co-production of and participation in research processes can take place. Here we will discuss the advantages of the proposed approach over other stakeholder analysis approaches, identify its strengths and weaknesses, provide an interpretation of the archetypes and show how the stratification can be applied in research processes.

### Advantages over other approaches

Previous approaches for determining ‘the right’ stakeholders include an analysis along a matrix of power and interest, power and influence, or power, legitimacy, and urgency (Buanes et al. 2004). The selection of the dimensions of such a matrix (e.g., power, interest, influence, or urgency) depends on the purpose of the analysis, what type of information is most relevant to the objective of the project, and for the uptake and implementation of stakeholders (European Commission et al. 2018). However, some of these dimensions are difficult to evaluate, e.g., how to evaluate the interest or urgency of an organisation in relation to other organisations. In this paper, the use of an indicator-based framework evaluated the overall relevance of stakeholders in the SES relative to climate change and adaptation governance objectives. In this way, we not only identify the ‘loud voices’, e.g., the ones with high power, influence, and interest, but also these organisations that are highly vulnerable to climate change, but with a low agency.

Furthermore, the organisation archetypes identified in this paper are conceptually equivalent to the different stakeholder roles within a complex system that has been previously and differently proposed (Goodman et al. 2017; Lyon et al. 2020). Goodman et al. (2017) proposed stakeholder activities and roles such as Stimulator, Initiator, Broker, Legitimator etc., while Lyon et al. (2020) defined stakeholder roles within a complex system and included roles such as Regulator, Decision-maker, Guardian, Owner etc. However, both these approaches require a much greater effort and access (compared to the stratification approach) to stakeholders to examine aspects such as interest, motivation, moral orientation, and transformational readiness that cannot be assessed quickly, and as part of a research project which is not solely focussed on stakeholders.

### Strengths and weaknesses of the approach

COVID-19 pandemic conditions created the need for greater involvement of expert evaluators but ideally, evaluation, or validation by a science-society expert group is preferable. The pandemic exponentially also increased the need for online engagement with stakeholders in various research activities. As such, the key strengths of this method were its simplicity and low resource needs (fast and efficient), with the possibility to remotely evaluate organisational agency. The indicator framework was flexible, and the research team adjusted the description of the indicators to fit the research objectives and the reason for which stakeholders would be engaged. The stratification method focussed the engagement process on key stakeholders and reduced project resources. This also reduced further engagement fatigue.

The weakness of the stratification includes a possible over-reliance on expert evaluation of the indicators. However, this can also be scaled according to time and resource availability to include more stakeholder input when the process allows, and less when engagement is challenging, such as during the COVID-19 pandemic, or when stakeholders become “fatigued” from engagement processes. The organisational complexity of some of the stakeholders may make the assessment of a single set of indicators problematic due to the inability (cost, time constraints) to assess individual units within an organisation. This is particularly relevant for large and multifunctional organisations such as local governments, and especially city governments (da Cruz et al. 2018). There is simply no single set of indicators with which to assess the system role of such large and complex organisations. It would be more appropriate to then assess individual functional units or line departments, as well as the overall administrative and political conditions that enable agency, i.e., the role of bureaucracy (Colenbrander and Bavinck 2017), or information flow within public authorities (Celliers et al. 2021a, b).

### Interpretation of archetypes and application in research processes

The five archetypes identified in this paper are relatable and easy to communicate the significance of the measures of agency in these statistical but also intuitive groups. It also identified the substantial differences in agency between these categories, and the imbalance between public entities such as local, provincial, and national government, and citizens, and civil society (business, industry, etc.) in general. The archetypes are broadly transferable to similar research settings.

The interpretation of the archetypes remained nuanced and did not simply represent boxes from which enough stakeholders should be drawn to participate. A high measure of agency meant that these stakeholders already make decisions and can change the system through policy or management or even physical means. However, a low measure of agency may have dual meanings. Low agency (and limited operation scale in the area of interest) may correspond to low interest or need to act in that place in time. As such, the engagement with these stakeholders in participatory processes is optional and their absence is not a loss of critical voices or opinion.

Low agency at the local scale may also mean greater vulnerability to change, e.g., extreme weather events. This may be particularly true for membership organisations, or associations where members themselves are vulnerable or limited in agency, such as local, subsistence fishers. The interpretation of low agency still requires a contextual understanding of organisations and their functions and operations within the area of interest, and relative to the research objectives. This will always require interpretation by the research team and the societal stakeholders themselves.

The stratification of stakeholders by agency proved useful in the further identification of the ‘right’ stakeholders for research on the SES in Algoa Bay. For example, a sub-sample of stakeholders was chosen for a network analysis of collaboration and knowledge exchange for climate change adaptation, only including those stakeholders that are locally based in Algoa Bay or have a specific mandate for local coastal governance (Rölfer et al., under review). Thus, the organisational archetype “on the margin” was excluded from this objective. Another example was the assessment of governance performance for climate change adaptation (Rölfer et al. 2022). In this case, representation from all archetypes was desired to integrate the perceptions of stakeholders from different levels of agency for scoring governance performance.

## Conclusion

In this paper, we developed and tested a methodology with which to stratify stakeholders and to understand their strategic and functional roles in a coastal social-ecological system. The organisational assessment and the statistical identification of archetypes were chosen for its flexibility (in terms of redefining indicators, selection of expert or stakeholder evaluators, remotely executed) but also for the convenience and relative speed with which the research team could develop a more nuanced understanding of the stakeholder and organisational landscape of a coastal SES such as Algoa Bay. This is particularly important for climate adaptation planning. In the approach described in this paper, we recommend including further examination of not only the assessment of agency in relation to an external (research) objective, but also an examination of the connectedness of organisations in a highly networked SES such as a coastal city. Further research, therefore, may include linking the organisational archetypes to a Social Network Analysis to disentangle the role of stakeholder groups with high agency in empowering and supporting stakeholder groups with lower agency, in the context of climate change adaptation.

## Supplementary Information

Below is the link to the electronic supplementary material.

Supplementary file1 (PDF 553 kb)
