# Supplementary material for: Stratification of stakeholders for participation in the governance of coastal social-ecological systems
Source: Ambio. 2023 Mar 23;52(9):1418–30. doi: 10.1007/s13280-023-01844-1 (PMC10035481; doi:10.1007/s13280-023-01844-1)
Supplement: Supplementary file 1 — Supplementary file1 (PDF 553 kb) [file 13280_2023_1844_MOESM1_ESM.pdf]

Ambio

Electronic Supplementary Information

This supplementary information has not been peer reviewed.

Title: **Stratification of stakeholder for participation in the governance of coastal social-ecological systems**

Authors: Louis Celliers L, Lena Rölfer, Nina Rivers, Sergio Rosendo, Meredith Fernandes, Bernadette Snow, María Mãnez Costa

Supplementary Table 1: A summary of the scoring system devised to evaluate the SCALE at which institutions operate within the coastal and marine management landscape of Algoa Bay.

#	Indicator	Evaluation	Score	Description
S1	Spatial scale of institutional operation (physical presence) in Algoa Bay	Yes	1	The institution has a **physical presence** in the Algoa Bay.
		No	0	The institution has **no physical presence** in the Algoa Bay
S2	Institutional mandate to achieve management objectives in Algoa Bay (operational scale)	Algoa Bay	3	The institution focusses on achieving management objectives **in the entire** Algoa Bay (across ocean and coastal boundaries)
		Parts of Algoa Bay	2	The institution focusses on achieving management objectives **ONLY in parts** of Algoa Bay (either ocean or coastal, or parts therefore)
		Beyond Algoa Bay	1	The institution focusses on achieving management objectives **beyond** Algoa Bay, but also including Algoa Bay (across ocean and coastal boundaries)
S3	Institutional representivity or constituency (membership or headcount)	National government	6	The institution is represented by the national voting constituency (membership or majority)
		Provincial government	5	The institution is represented by the provincial voting majority
		Local government	4	The institution is represented by the local municipal voting majority
		Large community (neighbourhood, club, NGO, forum, etc)	3	The institution is represented by a large (more than 1000 individuals) Algoa Bay-specific community, members or interest
		Medium-sized community (neighbourhood, club, NGO, forum, etc)	2	The institution is represented by a medium sized (100-999 individuals) Algoa Bay-specific community, members or interest
		Small community (neighbourhood, club, NGO, forum, etc)	1	The institution is represented by a small (less than 100 individuals) Algoa Bay-specific community, members or interest

Supplementary Table 2: A summary of the scoring system devised to evaluate the POWER potential of institutions that operate within the coastal and marine management landscape of Algoa Bay

#	Indicator	Evaluation	Score	Description
P1	Executive powers—Promulgate and cause to enforce legislation	Yes	3	The institution has and regularly uses executive powers
		Sometimes	2	The institution sometimes has executive powers
		Advise	1	The institution normally provides advice to agencies with executive powers
		No	0	The institution has no executive powers
P2	Legislative powers—Draft and set in motion the promulgation of legislation	Yes	3	The institution has and regularly uses legislative powers
		Sometimes	2	The institution sometimes has legislative powers
		Advise	1	The institution normally provides advice to agencies with legislative power
		No	0	The institution has no legislative powers
P3	Political relevance—The extent to which the actor has a political role or political influence to play in policy issues of Algoa Bay.	High	2	The institution has high political relevance in Algoa Bay or parts thereof.
		Low	1	The institution has low political relevance in Algoa Bay or parts thereof
		Neutral	0	The institution is politically neutral in Algoa Bay or parts thereof
P4	Moral suasion—The extent to which the actor can exercise its moral authority and status to harness public opinion and influence decision making on any given coastal issue. (The opposite of political relevance.)	High	2	The institution often exercises its authority and status to harness public opinion to achieve its goals in Algoa Bay or parts thereof
		Low	1	The institution sometimes exercises its authority and status to harness public opinion to achieve its goals in Algoa Bay or parts thereof
		None	0	The institution never exercises its authority and status to harness public opinion to achieve its goals in Algoa Bay or parts thereof
P5	Enforcement role (level at which an institution can effect the compliance with legal and management instruments).	Prosecute/monitor/arrest/summons	2	The institution is directly involved with monitoring, fines, summons, arrests, prosecutions in court of law
		Official reporting (as part of workflow)	1	The institution can cause a permit to be withdrawn
		None	0	The institution has no enforcement role

Supplementary Table 3: A summary of the scoring system devised to evaluate the RESOURCES that institutions have at their disposal within the coastal and marine management landscape of Algoa Bay

#	Indicator	Evaluation	Score	Description
R1	Human capacity—Staff numbers, skill and knowledge to affect objectives or initiatives in Algoa Bay. (it's not about large number of staff across the country but in the Bay). Or you may have low skill so maybe you have	Exceptional capacity for action in Algoa Bay	3	The institution has many staff with high skill and knowledge to implement actions **in Algoa Bay** or parts thereof
		Adequate capacity for action in Algoa Bay	2	The institution has many staff and low skill and knowledge or few staff but with high skill and knowledge to implement actions in Algoa Bay or parts thereof
		Limited capacity for action in Algoa Bay	1	The institution has a few staff and low skill and knowledge to implement actions in Algoa Bay or parts thereof
		No capacity for action	0	The institution has low numbers of staff and no skill and knowledge to implement actions in Algoa Bay or parts thereof
R2	Financial—Funding dedicated to achieving management objectives or initiatives linked to ABM in Algoa Bay. The ability of institutions to convert financial resources to actions, activities, and projects to the benefit of achieving (societal) management objectives.	Large or substantial ability to commit financial resources	2	**Substantial funding** to dedicate to projects, initiatives, activities to achieve management objectives of ABMs, most often by **public sector, NGOs etc**, but excluding for commercial or financial gain in the complete or large parts of the Algoa Bay. This excludes operational funding, e.g., salaries and other overhead costs except where those are dedicated to Algoa Bay.
		Limited or low ability to commit financial resources	1	**Limited or no funding** to dedicate to projects, initiatives, activities to achieve management objectives of Algoa Bay, most often by **public sector, NGOs** (shark conservancy for beach clean-up can commit time and effort but not huge financial resources), but excluding for commercial or financial gain in the complete or large parts of the Algoa Bay. This excludes operational funding, e.g., salaries and other overhead costs except where those are dedicated to Algoa Bay. Funding committed are minor
		Funding committed for self/shareholder-interest for profit.	0	Funding committed/ dedicated to projects, initiatives, activities to achieve management or financial objectives of ABMs primarily for **commercial or financial gain** in smaller parts of the Algoa Bay. Benefit is mainly for **self or shareholders**
R3	Infrastructure and material goods—Conversion of financial and human resources to support institutional objectives or initiatives in the Algoa Bay.	Good	2	The institution has a good infrastructure with which to contribute to implementation of objectives in Algoa Bay or parts thereof. The infrastructure is in service of Algoa Bay objectives. Offices, laboratories, specialized vehicles, specialized monitoring.
		Limited	1	The institution has a limited infrastructure with which to contribute to implementation of objectives in Algoa Bay or parts thereof. Mainly focusing on business or commercial or membership objectives with infrastructure for that purpose. Mainly formal offices with some specialized equipment mainly for own purposes
		None	0	The institution has no infrastructure with which to contribute to implementation of objectives in Algoa Bay or parts thereof. No local offices, or any material goods in the Algoa Bay.

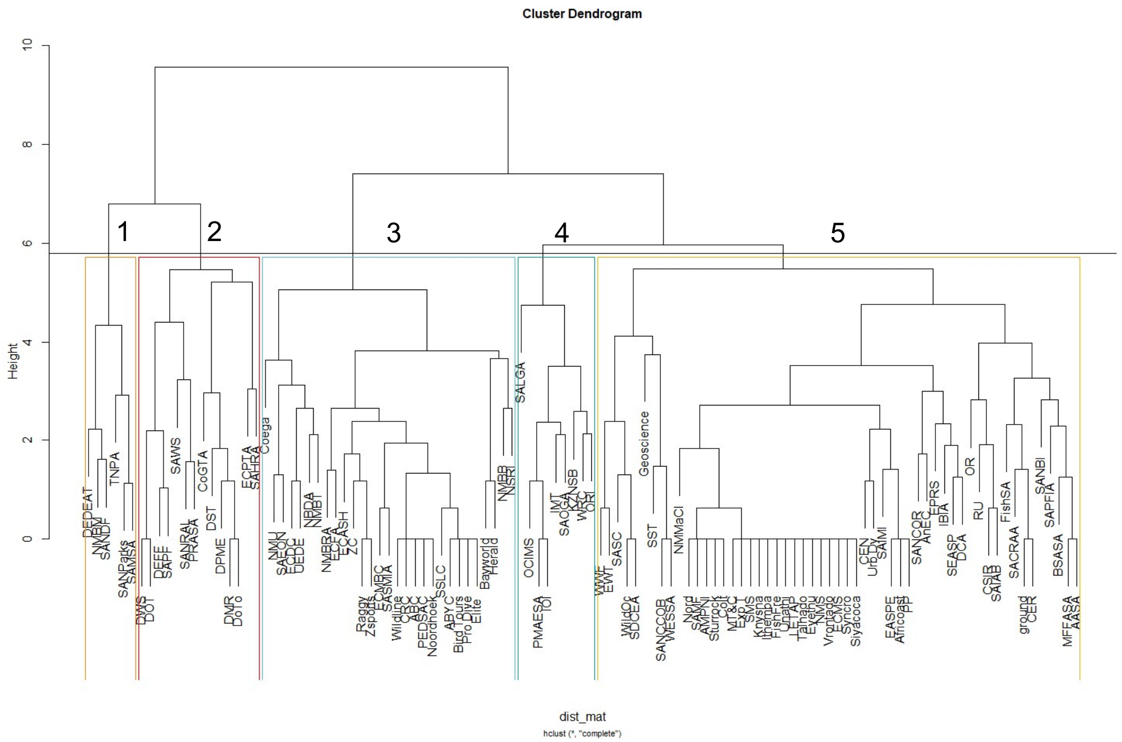

Supplementary Figure 1. Dendrogram visualizing the hierarchical agglomerative clustering of organisations involved in aspects of ocean and coastal governance of the coastal social-ecological system of Algoa Bay, South Africa, into five groups (1-5) using the stratification method described the paper.
